# Improving the science and evidence base of disaster response: a policy research study

**DOI:** 10.1186/s12913-019-4102-5

**Published:** 2019-05-02

**Authors:** Irene Anne Jillson, Michael Clarke, Claire Allen, Stephen Waller, Tracey Koehlmoos, William Mumford, Jeroen Jansen, Keith McKay, Alexandra Trant

**Affiliations:** 1 Georgetown University Medical Center, Department of Family Medicine, Building D, Room 234, 4000 Reservoir Road NW, Washington DC, 20057 USA; 2 Northern Ireland Methodology Hub, Centre for Public Health, Royal Hospitals, Queen’s University Belfast, Grovesnor Road, Belfast, BT12 6BJ UK; 3 Evidence Aid, Oxford, OX2 6GG UK; 4 Global Health and Surgery, Department of Preventive Medicine and Biostatistics, Uniformed Services University of Health Sciences, Bethesda, MD 20814 USA; 5 Department of Preventive Medicine and Biostatistics, Uniformed Services University of the Health Sciences, Bethesda, 20814 USA; 6 Gyeongsangnam-do, South Korea; 7 School of Nursing and Health Studies, Georgetown University, Washington, D.C., 20057 USA; 8 Trinity College Dublin, School of Natural Sciences, Dublin, D02 FD37 Republic of Ireland

**Keywords:** Disaster response, Humanitarian crises, Evidence-based disaster response, Ethical, legal, social issues

## Abstract

**Background:**

In order to elicit the knowledge, experience, and attitudes of individuals involved in disaster response with regard to evidence-based best practices, Evidence Aid and its institutional partners, Georgetown University and the Uniformed Services University of the Health Sciences, carried out a Policy Delphi study in 2015–2016.

**Methods:**

Purposive and snowball methods were used to select study participants. The Delphi study comprised two rounds of iterative questions, with the questionnaires completed online. In addition, participants at the Evidence Aid conference in November 2016 discussed the findings in focus groups. Excel was used to analyze the quantitative data and Glaser and Strauss (1967) to analyze the qualitative data.

**Results:**

Thirty-six participants responded to the first round of the study, 165 responded to the second round, and 30 participated in the focus group discussions. The salient findings include 1) ensuring that all key stakeholders are engaged in planning for and responding to disasters in a collaborative, coordinated manner—including local community members; 2) using, insofar as possible, evidence-based responses; 3) increasing and strengthening research to ensure that such data are available; and 4) addressing ethical, legal and social issues throughout the planning, immediate response, and post-disaster periods.

**Conclusions:**

Recent humanitarian disasters, due to natural and man-made hazards or a combination of the two, reinforce the need for more effective, efficient, humane responses at the local, national and international levels. This study has yielded findings that can be used to strengthen planning and response by taking into account, where possible, evidence based on research that has been carried out with the engagement of community members and with support by key stakeholders. The most effective means of facilitating the development and implementation of consistent, coordinated policies and practices might be for the United Nations Office for Disaster Risk Reduction to take the lead in engaging key organizations in the required discussions and collaborations.

**Electronic supplementary material:**

The online version of this article (10.1186/s12913-019-4102-5) contains supplementary material, which is available to authorized users.

## Background

Disaster planning and response has been largely based on long-standing practices, notwithstanding the fact that for more than a decade there have been calls for evidence-based practices (e.g. [1]). The patchwork of practices and preferences is often anecdotal, based on the history and perceptions of the host country’s leadership, non-governmental organizations (NGOs), and external aid organizations, rather than on the use of evidence-based interventions and practices. In an environment that increasingly recognizes the importance of accountability and the need to base actions on evidence, this raises the question of how practices that are supported by science and the main gaps in disaster response can be identified [2].

We investigated this broad question using the Policy Delphi technique, a policy analysis method designed to engage groups of experts (with experts being broadly defined) in dialogue regarding a single issue or multiple issues related to a particular topic. The Policy Delphi approach entails a series of rounds of iterative, structured dialogues (through mail or e-mail, questionnaires, meetings or a combination of these), with each building on the other. Initially, questions are posed and the respondents answer anonymously. The responses are then summarized and used as a basis for the questions posed in the succeeding rounds. Questions may be added to move toward a rich and substantive dialogue in the study.

Policy Delphi has been widely used in disaster management since the early 1970s, when it was first developed by the US Office of Emergency Preparedness. In contrast to the traditional Delphi approach, it does not seek consensus, but rather explores alternatives and their implications. In the health field, Policy Delphi has been used to address ethical, legal and social issues in biomedical and behavioral research, to forecast medical technological developments, to consider drug abuse policy, to plan for organizational and professional priorities, to develop national health priorities, and, at the community level, to engage communities in developing local health systems plans (e.g. [3–5]).

The purpose of this study was to engage a wide range of key stakeholders in dialogue regarding various elements of disaster response, including, for example, evidence for best practice, approaches to improving investment with systematic review analysis of evidence, identification of gaps in the evidence base; and factors that impact on effective disaster response. It builds on earlier needs assessment and priority setting exercises by Evidence Aid [6, 7], which is an international organization registered as a charity in the United Kingdom, dedicated to strengthening decision making in the disaster sector through the use of reliable and robust evidence [8, 9].

## Methods

This Policy Delphi comprised two rounds, with the first and second being completed online using SurveyMonkey. In addition, participants at the Evidence Aid conference in November 2016, in Washington DC, engaged in focus groups to discuss the findings. The Policy Delphi was designed collaboratively by the planning group for the study, which comprised individuals from Evidence Aid, Georgetown University (GU) and the Uniformed Services University of the Health Sciences. The GU Institutional Review Boards (IRB) approved the study, and GU disseminated the two rounds of questionnaires through SurveyMonkey, conducted the analysis, and served as the location for the Evidence Aid conference. The broad research questions were:

1. To what extent is evidence for best practices in disaster response available to a wide range of stakeholders?
2. To what extent is Cochrane-style (systematic review) analysis used to assess evidence for best practices in disaster response?
3. What are the most effective approaches to improving the cost-effectiveness of investments in disaster response?
4. How can the ethical, legal and social issues related to disaster response decision-making be most effectively addressed?
5. What are the factors that impact on effective disaster response decision-making?

Participants were selected by the study-planning group using a purposive sampling method; that is, we identified individuals who had published in the field of disaster/humanitarian response, who were public officials or representatives of NGOs engaged in the field, or who had participated in Evidence Aid conferences or other events related to disaster/humanitarian response. For the first round, 135 individuals were selected as potential respondents. For the second round, 237 potential respondents were selected, including the original 135 to whom the first round was sent.

An invitation email that included an informed consent script was sent to the initial 135 potential respondents, with a link to the questionnaire. The first-round, semi-structured questionnaire which was developed for this project (Additional file 1) comprised 24 questions in four categories: a) demographics; b) nature and quality of research-based evidence for disaster response; c) social returns of investments in disaster response; and d) effectiveness of current efforts in disaster response. Thirty-six individuals responded, a response rate of 27%. The second round of the Policy Delphi was disseminated by email (which included an invitation letter and informed consent script) to the 237 individuals who had been identified, with links to the round two questionnaire and the report of the first round. The second-round, semi-structured questionnaire, which was developed for this project (Additional file 2), comprised 20 questions in three categories: a) demographics; b) evidence for best practices in disaster planning and response; and c) effectiveness of current efforts in disaster response. These questions were based primarily on the results of the first round. Over two-thirds of the sample (165, 70%) responded to the second round. Thirty individuals who attended the Evidence Aid conference on November 16–18, 2016 participated in focus groups to discuss key questions related to the findings.

## Results

### Respondent professional demographics

Respondents to the first and second rounds of the Policy Delphi had similar professional demographic characteristics. Most respondents (37% in the first round and 52% in the second) reported working for an institution other than those listed (e.g., international other than development, scientific research, diplomatic corps). Nearly one-third (31%) of respondents in the first round reported working primarily at an academic institution or university, while 25% of respondents did so in the second round. Other respondents described their primary place of work as being involved in international development (11 and 6%), private sector non-profit organizations, other than development assistance (11% in both the first and second rounds), national government work generally (11 and 15%), government agencies involved in the provision of or support for international aid or development projects (9 and 1.3%), or private sector non-profit development assistance (9%). The categories mentioned by less than 9% (i.e., 3 respondents) but at least two were national government, health agency and national government other. In both the first and second round, some respondents indicated multiple roles.

When asked to specify their role in the organization with which they worked, 35% of first round and just 11% of second round respondents described themselves as researchers; 29 and 12% as professors or teachers; 21 and 13% as consultants or advisors; 18 and 28% as being involved in either administration or management; and 12 and 10% as clinicians. Nearly one-fifth (18%) in the first round and more than one-fourth (28%) in the second round selected the “Other” option, with the self-identified descriptions being primarily the same in both rounds: program manager/emergency manager, technical specialist, director of research, public health program coordinator, science and technology adviser.

There was a difference between the two rounds in geographic location of the respondents: in the first round, one-fourth were based in the United Kingdom (25%), followed by the United States (17%), India (8%), and Switzerland (8%). In the second round, most reported being based in the United States (77%) followed by the United Kingdom (4%). Eleven participants did not respond to this question in the second round.

### Summary of findings by round and focus group

#### Round one

There was agreement among the 36 respondents that international and multi-national organizations, as well as national governments and NGOs could strengthen their response to disasters by using available evidence, and that systematic reviews should be used to synthesize evidence of disaster response effectiveness. Most believed that, although research-based evidence is preferable, for the most part it is ‘best practice’ information that serves currently as the basis for disaster responses determined by decision-makers and practitioners. Most respondents also believed that improvements in co-operation/co-ordination among disaster relief agencies in the collection, analysis and dissemination of data and information regarding disaster response would improve disaster response. Indeed, co-operation and co-ordination among all key stakeholders was a consistent theme through the first round responses, with respondents noting that this would contribute to improving social return on investments in disaster response, for example. Respondents believed that, although there are major impediments to effective and equitable disaster response that derive from both the country in which the disaster occurred (e.g., economic and political factors) and external factors (e.g., global economic influence and post-colonial linkages), these impediments can be addressed through the use of evidence-based policymaking and specific disaster-responses.

#### Round two

The 165 respondents to the second round consistently identified three factors that facilitate the development of ‘best practices’ for disaster planning and response: 1) increased accessibility to scientific evidence; 2) improved communication among stakeholders; and 3) engagement of multiple beneficiaries in the decision-making process. Most respondents believed that policy and decision-makers need to be educated about research to effectively implement evidence-based policy. Many respondents believed that accessibility to information at all levels can be challenging. They also believed that overcoming communication and financial barriers as well as problems with distribution of information can improve accessibility of information to ensure evidence-based decision-making. Furthermore, co-operation and co-ordination among all key stakeholders and beneficiaries was a consistent theme through the responses.

#### Focus group discussions

Participants suggested that policy makers should focus on dissemination of evidence related to disaster planning and response, communicating data, and creating a clear chain of command. Funding for research regarding disaster response and strengthened systems was also a concern. This would necessitate a clear plan and strengthening the capacity of all those involved, based on evidence. Participants identified lack of co-ordination between and among NGOs, stakeholders, local authorities and donors as both a key issue and a need that must be addressed with evidence insofar as possible. This included co-ordination regarding 1) assessment of needs and evidence-based practices (and appropriate analysis and dissemination of findings); 2) strategic planning for disasters; and 3) responses and follow-up assessments. Educating the stakeholders on the evidence-based responses and creating a universal standard (or models) were also essential to better planning for and responding to disasters, as was promoting a free flow of information to all, in particular populations affected by disasters.

### Results by topic areas addressed

In the following sections, the results of the two rounds of the Policy Delphi and the focus groups are presented by the topic areas addressed:

- Use of Research-based Evidence to Strengthen Disaster Responses;
- Perceptions of Ways that Ethical, Legal and Social Issues Related to Disaster Response Decision-Making be Most Effectively Addressed;
- Factors that Impact Effective Disaster Response Decision-Making; and
- Challenges and Approaches to Overcoming the Challenges of the Expected Outcomes of the United Nations International Strategy for Disaster Reduction (UNISDR) Road Map for Implementation of Sendai

### Use of research-based evidence to strengthen disaster responses

Overall, respondents agreed (mean: 4.0–4.5 out of maximum of 6) with the following statements:

- The nature of the evidence for disaster response is primarily best practice information rather than research-based evidence.
- It is possible for national governments to achieve better social return on investment in disaster response by implementing actions or interventions that are based on research evidence.
- It is possible for international and multinational organizations to better achieve social return on investment in disaster response.
- Cochrane-style systematic reviews should be used in a standardized way to synthesize evidence to inform contextually specific evidence of effectiveness in disaster response.

The analysis of answers to questions related to research-based evidence to strengthen disaster responses yielded four themes: 1) availability of evidence for best practices in disaster response; 2) most effective ways to improve the applicability of research-based evidence for pre- and post-disaster planning; 3) most effective ways to ensure that ‘best practices’ inform the design of disaster-related research to prove evidence of effectiveness; and 4) improving the applicability of research-based evidence for pre- and post-disaster planning. If a question was posed in only one round, this is mentioned in the findings. Otherwise, comparisons between rounds are presented.

#### Availability of evidence for best practices in disaster response

Individual participants mentioned increased research- and evidence-based case studies, more specific and thoroughly researched interventions, and universal access to this information as examples of how this could be accomplished. Responses about the nature and quality of research-based evidence for disaster response suggested that the disaster response community may be divided regarding their perceptions of the reliability of research based-evidence as the basis for strategic planning by national governments or international development agencies.

Of the 36 respondents to Round 1, 21 (58%) either completely or strongly agreed with the statement that ‘[t]he nature of disaster response is primarily ‘best practice’ information rather than research-based evidence’ (58%). The remaining participants ‘somewhat agreed’ (six participants), ‘somewhat disagreed’ (two participants), or ‘completely disagreed’ (one participant) with the statement. Three participants did not provide a response. An overall mean rating of 5 suggests that the respondents believe that best practice information rather than research comprises the basis of ‘evidence’ currently used in disaster response strategic efforts and planning. There was no consensus regarding the statement that research-based evidence for disaster response is sufficiently reliable to warrant its use as the basis for strategic planning by national governments. Indeed, one respondent suggested that international agencies should use evidence “*only when it has been peer reviewed, published, and critiqued*”.

#### Most effective ways to improve the applicability of research-based evidence for pre- and post-disaster planning

Responses suggested that the disaster response community may be divided regarding their perceptions of the reliability of research-based evidence as the basis for strategic planning by either national governments or international development agencies.

Of the 165 respondents who participated in the second round, 109 (83%) thought that one of the most effective methods to address this issue was for potential users of research related to pre-disaster planning (i.e. policy makers, planners, emergency response personnel) to be involved in the design and/or implementation of the research. This response was also selected as one of the most effective ways to improve applicability of research-based evidence for post-disaster planning by 104 (80%) respondents. Ensuring that research related to planning is decision-linked was chosen by 74 (57%) respondents for pre-disaster planning and by 70 (54%) respondents for post-disaster planning. Ensuring that comprehensive research is carried out (i.e. addressing socio-economic, life sciences, governance, and other aspects) was selected by 77 (59%) respondents as another effective way to improve the applicability of research-based evidence for post-disaster planning and by 65 (50%) respondents for pre-disaster planning.

Open-ended responses to this item suggest both an emphasis on collaboration with various parties and the use of gap analyses to identity areas of vulnerability for pre-disaster planning. 45 (34%) participants selected and wrote that multiple research methods should be used for pre-disaster planning, with one stating, “*When talking about evidence, we need to make sure that it is context-specific*” for pre-disaster planning.

Respondents’ suggestions for post-disaster planning emphasized ways to disseminate information and the importance of using evidence-based research to improve policy. Ensuring that the research is guided by priority setting or gap map exercises with a substantial methodology was a goal chosen by 48 (37%) participants. Many respondents mentioned the importance of having researchers ‘directly engage and actively participate with ‘the local community’, ‘subject matter experts’, policy makers, and planners to improve the applicability of research-based evidence for post-disaster planning’.

#### Most effective ways to ensure that ‘best practices’ inform the design of disaster-related research to prove evidence of effectiveness

Of the 66 respondents for this question, three quarters (76%) chose ‘ensuring that research-based evidence is available at no or minimal cost’ as one of the most effective ways to ensure that ‘best practices’ inform the design of disaster-related research to prove evidence of effectiveness’. More than half (55%) also chose the dissemination of ‘best practice information’ related to disaster planning and response through international and national associations of related professionals (e.g., World Medical Association, International Nurses’ Association, International Association of Emergency Managers) only if that information had been critically reviewed by an approved body. Nearly half the respondents (48%) selected ‘dissemination of ‘best practice information’ related to disaster planning and response through a central, global network only if they have been critically reviewed by an approved body.’ One respondent suggested that, rather than focusing on disseminating information, there should be an emphasis to “*work with one group to make a good example of use of best practice information, and then publish the case study*”.

As a follow-up question, respondents were asked whether evidence regarding disaster planning and response should be used only if it is peer reviewed, published and critiqued. Many (42%) indicated that all those processes should occur if the evidence is going to be used. Others (29%) indicated that it should be peer reviewed, while 22% of participants chose that it should always be published and 20% that it should be critiqued. Ten respondents indicated that while published and peer-reviewed literature is ideal, it is important to consider other information as well. One respondent said that they would “*prefer an open discussion since information, whether proven valid or not, may spur innovations into disaster and emergency planning, response and recovery*”. One respondent suggested that evidence-based data should include qualitative information, which “*should always be a great part of the disaster reviews*”. Another wrote that using standardized reviews would help “*regional governments to prioritize the focus of systematic review. I also believe that there is still a dearth of peer-reviewed evidence relating to disaster response* [and] *we need to be more open and find better ways of synthesizing grey literature*”.

### Engaging potential beneficiaries in research related to disaster planning and responses

Respondents were asked to describe how potential beneficiaries (i.e. policy makers, planners, responders, government officials, other public employees, local NGOs, individuals and populations affected, and members of the general public) should be engaged in research related to disaster planning and response while considering the lifespan of research that runs from the selection of priorities through designing and implementing the research to disseminating its findings. All 54 respondents who answered this question suggested that policy makers, planners, and government officials should be engaged in the research process. Sixteen respondents emphasized the importance of making sure that policy makers are educated about research findings and adequately prepared to make decisions and to develop policies. Seven respondents mentioned that policy makers should have a role in ensuring funding allocation for research related to disaster planning. Suggestions about the role of government officials focused on education and providing input in regards to funding, policy, and advocacy. Ten respondents mentioned that planners should determine the practicality of plans or policies. One respondent said that planners should “*ensure that the policies established are aligned with practicality on the ground*”.

Most (39 of 40) respondents who answered this question suggested that other public employees and local NGOs ‘should be informed of the results in ways that are relevant to them’. Seventeen of 40 respondents suggested that other public employees be educated in results and plans when it is applicable to their role. Similar suggestions were offered for local NGOs. Ten of 41 respondents mentioned that local NGOs should be involved in research, whether through participation in the study or designing the research if that would be relevant to their role.

On the other hand, respondents suggested that individuals and populations affected by disasters should be engaged in the research process as informants and consultants in regards to priority planning and implementation. Eighteen of 42 respondents suggested that those affected should be included as stakeholders through various parts of the process, such as the development of the research question, data collection, and the design and implementation of the project itself. One respondent said that those affected be involved in generating “*open forums to help guide research topics*”. Another respondent stated that all the listed potential beneficiaries of disaster response “*need to have active outreach for their input, however, not all need to be invited into the process from the very beginning*” to suggest a time-appropriate engagement of each stakeholder. Other suggestions included professional data collection organizations, healthcare organizations, and academic institutions as sources of research data and information. Two respondents also suggested engaging the media.

When asked how the potential beneficiaries of disaster-related research should be engaged in applying research findings related to disaster planning and response to strengthening their capacity to plan for and respond to disasters, similar responses were given for all potential beneficiaries. Respondents placed emphasis on political influence for planners (15), policy makers (13), and government officials (8). Again, respondents mentioned engaging public employees and local government officials if it is applicable to their role. Those affected by disaster were also deemed to have a valid role as research informants and consultants.

### Practical use of evidence for best practice in disaster response: social returns on response

Nine questions elicited beliefs concerning social returns on investment in disaster response and the potential of evidence-based research to improve upon them for national governments, international and multinational organizations, and local NGOs. One of these questions invited respondents to rate their agreement with the statement that it is possible for national governments to achieve better social return on investment in disaster response by implementing actions or interventions that are based on research evidence. The average rating was 5 on the six-point scale, indicating that respondents believed that research-based evidence could improve social returns on investments by national governments in disaster response efforts. When asked to provide up to three examples of how this could be accomplished by national governments, 11 respondents mentioned strengthening research related to evidence-based practice in disaster responses, including for example, providing additional funding, training researchers, and conducting specific types of research. The latter include research related to the Disability Adjusted Life Years (DALYs) or Quality Adjusted Life Years (QALYs) of interventions. Few respondents indicated that they believe that national governments could not improve return on investment by research-based interventions. The reasons provided were wide-ranging, including the belief that disaster responses are essentially ‘common sense’ and should not be ‘academic’, that the evidence is ‘just not there’ and that the decision-making process is inherently political. One respondent suggested that the full support of the United Nations is required.

Respondents similarly agreed that international non-profit organizations could improve social returns on investments by using research-based evidence for disaster response investments (mean rating: 4.71). With respect to the potential for non-profit organizations to improve evidence-based responses, most respondents (10 of 15) mentioned co-operation/co-ordination. Additional and/or improved evaluation and research related to NGO responses were recommended by eight respondents, and strengthened implementation of disaster responses by seven, with suggestions including investments in disaster mitigation and in community-level actions.

A similar question addressed the potential for research-based evidence to produce greater social returns on investments in disaster response for international organizations. There was relatively strong agreement with this statement as well. Co-ordination and co-operation were also the primary ways that respondents suggested that international and multinational organizations can improve disaster response, suggested by 28 respondents, including, for example, ‘strengthening partnerships between academicians, civil society and government bodies’. Eleven respondents suggested improvement in the use of evidence and in planning for disaster response. Specific examples included flexibility in assistance such as unconditional cash transfers that allow for beneficiary choice. Evaluating and conducting research regarding disaster response to yield evidence regarding best practices was suggested by 10 respondents. Other suggestions included focusing on the needs of vulnerable populations. Only one respondent did not believe that it is possible for international organizations to improve their disaster response, with the explanation that “*agendas present within multiple multi-national organizations may hinder benefits to all social groups*”.

Twenty-five respondents answered the question asking for up to three ways that knowledge and evidence derived from research could be used to inform decision-making in disaster response. Most of these suggested improvements in evaluation/research related to disaster response, with respect to the quality of the studies, an improved focus on decision-linked, relevant questions/research, ensuring that the findings are accessible, or dissemination of the findings to decision-makers and engaging them in discourse regarding the findings. Nineteen respondents suggested improvements in the practical application of evidence, including for example, educating policy makers regarding evidence-based practices, ensuring visibility of the findings (e.g. conducting conferences and seminars among user groups), and linking domestic/national-level responses with international responses. Co-operation was mentioned by seven respondents specifically and was a theme through many of the other suggestions. One respondent commented that: “*A survey that focuses on response is missing the point that most effective measures to reduce health consequences lie in prevention and building the capacity of countries, communities and the international community before an emergency and disaster*”.

### Perceptions of ways that ethical, legal and social issues related to disaster response decision-making be most effectively addressed

#### Ethical

Sixteen respondents responded to the item related to ethical issues; six of these provided examples that related to triage of populations and areas that are affected. A few respondents (6) suggested that research should be used to more effectively address the ethical issues of triage. One respondent suggested that “*conducting research into whether or not different approaches to triage are better for victims and responders*”. Other respondents suggested improved infrastructure, greater public awareness, and reviews of financial reports of aid organizations to which contributions are made for the disaster response.

Five respondents commented on the ethical issues related to allocation of resources, with one noting the role of leadership in determining “*when to respond and who should respond from outside the country*”. Responses pertaining to ways that ethics has been adequately addressed varied. Five participants mentioned enhanced training in ethical decision-making as well as collaboration with bioethicists. One wrote that “*establishing crisis standards of care protocols*” is a way that the issue of resource allocation has been addressed.

#### Legal

Over half of the 18 respondents who addressed legal issues provided examples related to liability with respect to healthcare professionals who volunteer to help with disaster response and the safety of first responders. Other examples (4) mentioned were difficulties of consent related to quarantine, loss of identification, record keeping, and the use of human subjects in research. Responses (6) related to ways that the legal issue has been addressed include collaboration with international agencies, local public health organizations, as well as “*judicial authorities*”. Other participants (3) mentioned observing how individual states have addressed the issues of consent.

Suggestions for the ways legal issues can be addressed more effectively with improved evidence related to policy (4) and research (4). Some responses related to policy related to the legal and liability issues that could arise. One respondent stated that “*developing clear policies within international organizations*” is one way to address the issue of liability. Another offered that political will is key to developing evidence-based disaster preparedness plans. In regards to research, one respondent said that research related to liability should be on the “*legal responsibility in international disasters*”. In relation to the issue of consent, one respondent suggested that people being studied in research should be part of the design phase.

#### Social

Of the 13 social examples provided, five related to resource and economic disparities while three related to gender. A participant noted that the disparities should be addressed across countries and within communities. In regards to gender, one respondent explained that “*understanding gender roles* [can] *impact how societies respond to disasters*”. The suggestions for ways these issues have been addressed vary. Two responses related to the issue of gender involved research. One respondent specifically mentioned that “*qualitative research is at least identifying some of these issues and their importance in responses*”. Four responses related to collaboration that involved government officials, insurance companies, general community members, and religious organizations.

The responses for how social issues can be more effectively addressed through improved evidence varied. Four respondents discussed improved training and technology. For instance, one mentioned ensuring that “*we have the systems, technology and the scientific methods to deliver this global target*” of enhanced disaster response and preparedness. Another suggested “*inclusive and collaborative policies*” while another mentioned how the evidence could be used to “*address health or social issues identified in the research*”.

### Factors that impact effective disaster response decision-making

The first Delphi round included a request that respondents rank what they considered the most important five of 15 factors presented for consideration in terms of their impact on the effectiveness of disaster responses, with 1 being the most important factor, 2 the next most important factor, and so on. The five factors that received the total lowest scores—that is, that respondents considered the most important—were:

- Political Influence of the government in which the disaster occurred.
- Sociological trends in the country in which the disaster occurs.
- Post-colonial linkages between donor countries and countries affected by disasters.
- Global economic influences in the country in which the disaster occurs.
- Economic influences of the country in which the disaster occurs.

Respondents were invited to identify other factors that were not included in the list of 15 as the most important impact on effective disaster response decision-making. Six respondents suggested a total of 10 other factors. Three suggested accountability or capacity (at the local and national levels) as critical factors. The nature/magnitude of the disaster was mentioned by two respondents. Other factors mentioned were the clear message that scientists could use to inform policy and practice, and the economic and health impacts within the country in which the disaster occurs and those with trans-boundary impact as well.

Respondents were then asked to indicate, for each of the five factors that they considered the most important, whether they believed that each had a primarily positive, negative, or mixed impact on the effectiveness of disaster response. All the original list of 15 factors were selected by at least one respondent. For 12 of the 15 factors, most respondents indicated that the factor had both positive and negative impact. For three of the factors, the respondents believed that there was primarily positive impact. These are: political influences of non-profit organizations of the country in which the disaster occurs; international legal factors, including for example laws and regulations regarding local NGOs, reconstruction and engagement; and ethical factors, including international guidelines regarding allocation of resources related to disaster relief.

Respondents were also asked to suggest ways that the factors they identified as the most important could most effectively be addressed in the context of disaster relief. The suggestions of the 13 who responded to this request all centered on the need to improve policy-making and implementation of disaster response, including concerning evidence-based approaches. However, within these parameters the suggestions were wide-ranging, with a focus on improving the coordination among and responses by the donors (international agencies and non-profit organizations), and considering political will when planning for and implementing disaster response. One respondent suggested, “*Ensure early (ideally pre-disaster planning phase) outreach to establish alignment of political influences with research based...best practices*”. Referring to one aspect of the politics of disaster relief, one respondent suggested that “…*there is nothing ‘potential’ about competition for funding among international relief organizations. It’s real and potent*”. Creating and sustaining dialogue and ensuring accountability among the key actors were mentioned by most of these respondents. Ten respondents mentioned the need to strengthen the capacity of key stakeholders, from policy makers to emergency response personnel. One respondent specifically referred to the Sendai Framework for priorities.

The main suggestions for the most effective way for best practices and improved evidence to be used to help ensure *accountability* for disaster planning and response related to education (7) and evaluation (5). In terms of evaluation, a respondent suggested developing “*post intervention studies [that] can help provide feedback to the concerned authorities*”. Another said that “*If we had evidence on best practice in disaster response and planning - we could use this as a way to evaluate and thereby ensure accountability*”.

### Challenges and approaches to overcoming the challenges of the expected outcomes of the UNISDR road map for implementation of Sendai

The UNISDR Sendai Framework for Disaster Risk Reduction 2015–2030 was adopted at the Third UN World Conference in Sendai, Japan, in March 2015 with a goal of “The substantial reduction of disaster risk and losses in lives, livelihoods and health and in the economic, physical, social, cultural and environmental assets of persons, businesses, communities and countries” [10]. This roadmap has become a major basis for collaborative planning within and among nations. Respondents to Delphi Round Two were presented with the seven expected outcomes listed in the UNISDR Road Map for Implementation of the Sendai Framework. These expected outcomes suggest how scientific data should be assessed and applied to reduce risk of disasters. Thirty-three respondents provided responses for this section. They were asked to identify one challenge about the expected outcome and to suggest a possible solution to overcome that challenge. The expected outcomes suggest how scientific data should be assessed and applied to reduce risk of disasters. Thirty-three respondents provided responses for this section.

#### Assess and update the current state of data, scientific and local and indigenous knowledge and technical expertise availability on disaster risks reduction and fill the gaps with new knowledge

When asked to present a challenge with assessing and updating the current state of data, scientific, local and indigenous knowledge as well as technical expertise availability on disaster risks reduction and fill the gaps with new knowledge, some respondents (12) mentioned challenges in regards to reviewing the data and personnel to perform this review. These respondents suggested enhanced support and tools as a solution to issues with reviewing the data and personnel. One respondent suggested building “*capacity for knowledge creation and exchange through local/non-local and expert/non-expert partnerships and community involvement*” as a potential approach to enhance support in this area. Other issues mentioned by respondents related to access to information (5) and funding (4).

#### Synthesize, produce and disseminate scientific evidence in a timely and accessible manner that responds to the knowledge needs of policy-makers and practitioners

Most (18) respondents presented challenges that related to accessibility of science evidence in terms of applicability to specific countries, target audiences of the study reports, and the length of time to find and publish new data. Solutions to the challenges included “*provide other means of communication for dissemination of scientific evidence*,” “*prioritize knowledge needs based on the situation for policy makers*,” and “*translate into effective, non-technical information*”. The overall solutions suggested that needs-based data should take priority and that multiple groups of people should have access to and be able to comprehend the data.

#### Ensure that scientific data and information support are used in monitoring and reviewing progress towards disaster risk reduction and resilience building

Respondents (16) identified accessibility as a challenge in regards to ensuring that scientific data and information are used to monitor and review the progress towards disaster risk reduction and resilience building. The challenge of accessibility was defined in terms of ensuring all potential implementers and beneficiaries have access to the evidence as well as the ability to comprehend and implement it. A respondent described the challenge of accessibility as that “*key actors* [are] *unaware of scientific data/unable to keep abreast of new research*”. Participants presented solutions that pertained to enhanced dissemination of information and that focused on increasing involvement in the research and planning processes. Issues with reviewing the scientific data and information were mentioned by 13. One participant specifically asked, “*how do we verify if the data and information was used during a disaster?*” He/she suggested providing “*a reporting requirement that [specifies] which portions of the data and information were used and to what extent*” to reduce the risk of disasters. One respondent mentioned that participants should be ‘incentivized’ and another suggested “*acquiring passionate stakeholders in a dedicated committee*”.

#### Build capacity to ensure that all sectors and countries have access to, understand and can use scientific information for better informed decision-making

Most (18) respondents indicated that communication barriers would present a challenge to ensuring that all sectors and countries have access to, understand and can use scientific information for better informed decision making. Communication barriers included trust, language, and cultural differences. Some of these same respondents (8) also expressed that these communication barriers lead to a lack of evidence-based action. One participant indicated that a challenge is the “*lack of ability to interpret and practically apply scientific information*”. The solutions suggested for this challenge included generating ‘continued partnerships between researchers and emergency preparedness and response personnel’. Solutions to the problem of communication barriers varied. Some participants suggested forming a global consortium of information, to make the data available at a low cost, or to offer ‘specific financial and competence aid in this area,’ and another suggestion was to ensure that ‘research is truly designed to impact needs’.

#### Support a stronger involvement and use of science to inform policy- and decision-making within and across all sectors at all levels

Approximately one-third (9 of 29) of respondents mentioned issues with distribution of information to support a stronger involvement and use of science to inform policy- and decision-making within and across all sectors. The challenges with distribution of information included ‘getting the information out across all sectors,’ ‘the use of evidence at all levels,’ and the fact that ‘many participants do not have this knowledge and need to be informed’. Solutions to these specific challenges included academic training, implementing ‘more research related to [the] impact of different strategies,’ and incentivizing participants. Two participants also mentioned that ‘tradition and past practice have a strong resistance to change’ and that the ‘acceptance of science to develop policy and decision making’ pose ideological challenges that inhibit a stronger involvement and use of science in policy and decision-making. Solutions to these challenges included identifying examples of effective use of science in policy and decision through identifying ‘champions in various sectors who will advocate for evidence-based policy-making’ as well as using science-based policy related to indigenous populations as examples of how policies should or should not be implemented. Nearly half (13 of 29) of respondents presented solutions related to generating enhanced support of evidence-based policy through education and the incorporation of political contexts.

#### Provide scientific evidence to enable decision-making of policy options for investment and development planning vulnerable communities and locations

Challenges presented by respondents were varied and reflected many of the challenges mentioned above. Some (6 of 28) respondents selecting this UNISDR outcome mentioned that gaining evidence about vulnerable populations is a challenge. One respondent presented the solution of focusing “*on one or two areas of highest priority – lower the bar for scientific evidence, if possible*”. Other challenges included the view that “*vulnerable communities have few decision-making resources*” and that there is a “*lack of involvement of primary stakeholders, therefore a lack of evidence on what works for different vulnerable groups*”. Solutions for challenges related to these issues included increasing education efforts and encouraging involvement from stakeholders. Over half (16 of 29) of all respondents suggested education and enhanced engagement as solutions to the challenges to providing scientific evidence to enable decision-making of policy options for investment and development planning for vulnerable communities and locations.

#### Identify and respond to the needs of policy- and decision-makers at all levels for scientific data and information to strengthen preparedness, response and to “Build Back Better” in recovery, rehabilitation and reconstruction to reduce losses and impact on the most vulnerable communities and locations

The challenges to this UNISDR outcome that the respondents suggested varied, but 11 of the 27 respondents presented issues that related to a lack of understanding and accessibility of information. Other suggestions (10) indicated issues related to educating stakeholders, such as policy and decision makers, about the needs of vulnerable populations. Four respondents mentioned that funding would pose a problem to obtaining this outcome. Most (12) solutions suggested enhanced education and engagement. One solution included the importance of listening ‘to the needs of the policy- and decision makers, and craft the implementation of the scientific data and information to align with existing local/indigenous authorities’ goals’. Four mentioned involving the local community as a specific type of engagement.

### Co-ordination and co-operation among all stakeholders

Co-ordination was a principal theme in the first round, when most respondents (27) suggested that improvements in co-operation/co-ordination among disaster relief agencies in the collection, analysis and dissemination of data and information regarding disaster response would improve disaster response. Fewer responded that improvements in disaster response would come from improvements in funding for research to yield evidence (20) or increased use of evidence in planning and implementing such responses (9).

Over a third of respondents (11 of 29) respondents suggested methods that relate to communication among stakeholders, such as ‘open, honest communication’, the use of ‘pre-established communication channels’, and ‘congeniality and collaboration.’ Other responses (8) pertained to ensuring the involvement of all stakeholders. Some of these responses included “*involving them* [all stakeholders] *in research*”. Another respondent mentioned “*more inter-agency research*” within the private sector and community engagement for the public and private sectors.

When participants in the focus groups were asked how they would improve the engagement of stakeholders in pre- and post- disaster response and planning, they commented that it was critical to strengthen coordination of services between the NGOs, stakeholders, and local authorities, assessing the areas needing improvements, and proper analysis and dissemination of the data presented. One respondent said that “*if stakeholders know that other people are looking at their use of expenses… they will be more likely to respond in an evidence-based manner*”. Coordinating the services and ‘*identify(ing) the real key stakeholders’* and ‘*engaging (them to identify) what they want to know from research evidence… so that work on evidence is driven according to stakeholder priorities*’ were identified as ways to improve emergency response and preparedness.

Focus group participants were also asked what action they would take to improve pre- and post- disaster and emergency cooperation and coordination in key stakeholders. Their responses focused mainly on the need for communication between NGOs and stakeholders to ‘reduce (the) overlap of responsibilities’ and streamline the process in a more efficient manner. Additionally, the need to identify the proper chain of command of stakeholders is also a priority. ‘*Mapping stakeholders and understanding their area of expertise and capability (and bring) them together on specific areas of concern’* is crucial to making the response and preparedness actions more well-organized and effective. The need to collect and share the data in a manner that is ‘clear and transparent’ was seen as critical, as was educating stakeholders using evidence-based data and information and creating a universal standard for disaster planning and response.

## Discussion

There was agreement among respondents about the value of increased accessibility to scientific evidence, improved communication among stakeholders, and engagement of multiple beneficiaries to facilitate the development of evidence-based ‘best practices’ for disaster planning and response. Most believed that policy and decision-makers need to be educated about the research to effectively implement evidence-based policy. Many respondents believed that accessibility to information at all levels can be challenging and that overcoming communication and financial barriers, as well as problems with distribution of information, can improve accessibility of information to ensure evidence-based decision-making. Furthermore, cooperation and coordination among all key stakeholders and beneficiaries was a consistent theme.

International and multi-national organizations, as well as national governments and NGOs could strengthen their respective response to disasters by using available evidence, and Cochrane-style systematic reviews should be used to synthesize evidence of disaster response effectiveness. Most believed that, although research-based evidence is preferable, for the most part it is ‘best practice’ information that serves currently as ‘evidence’ on which decision-makers and practitioners create their disaster responses. Most respondents also believe that improvements in co-operation/co-ordination among disaster relief agencies in the collection, analysis and dissemination of data and information regarding disaster response would improve disaster response. Indeed, co-operation and co-ordination among all key stakeholders was a consistent theme through the responses, contributing to improving on social return on investments in disaster response. Respondents believe that, although there are major impediments to effective and equitable disaster response that derive from both the country in which the disaster occurred (e.g., economic and political factors) and external factors (e.g., global economic influence and post-colonial linkages), these impediments can be addressed by practicing evidence-based policy-making and specific disaster-responses.

To improve the use of evidence in pre-disaster and post-disaster planning and response, those responsible for disaster response planning and implementation must share information, increase the scientific and evidence-based foundation for disaster response and establish a mechanism to consolidate decision-making. To facilitate the sharing and use of evidence-based information, it should be presented in ‘lay’ language to increase the likelihood of broader use by a wide range of key stakeholders. Additionally, consideration should be given to creating and maintaining a central source of evidence-based policies, practices, and other data and information that can be made accessible to disparate groups through a wide range of mechanisms, including but not limited to the Internet. The use of evidence should become a habit in pre-disaster planning and post-disaster response, with decisions made at the central level being evidence-based. Furthermore, a mechanism for accountability is key to ensuring the scientific and evidence-based foundation. Consolidating decision-making allows information to be spread more rapidly, increasing accountability in evidence-based actions, and provides a focal point of command.

To improve the engagement of key stakeholders, pre-disaster and post-disaster planning and response, steps need to be taken to increase transparency, expand needs assessments, and encourage community participation. Transparency on the part of the government, NGOs, and other aid organizations increases access and accountability, making key stakeholders more willing to be engaged in the process. As an aspect of increasing transparency, expanding needs assessments engages stakeholders and improves the likelihood of success through raising knowledge of the actual needs of a community or region. A better understanding of actual needs will offer an opportunity for a more comprehensive and catered plan to the area experiencing a disaster. Furthermore, community participation will not only make those planning and responding to disasters more aware of actual needs, but also will help to ensure that those organizing or directly providing responses are more accessible and transparent to the community. In turn, this encourages active community participation in prevention of and responses to disasters.

Improving co-operation and co-ordination among key stakeholders in pre-disaster planning and post-disaster response is vital. To do so, steps must be taken to open dialogues among key stakeholders, make funding more fluid and adaptable, and encourage a set standard for disaster planning and response, be it by a hierarchy of command or metrics for accountability. Creating dialogue between and among stakeholders will allow for a better sense of each stakeholder’s strengths and abilities, as well as bring synergy to disaster planning and response. Dialogues can offer a map of stakeholders and their expertise. Flexible and adaptable funding allows for increased co-operation and co-ordination, allowing for the *right* stakeholder to be engaged in an aspect of disaster planning and/or response. A set standard or metric for disaster response will mitigate squabbles over jurisdiction and role, increasing synergy and co-ordination. An established standard will make language uniform and accessible, implement a chain-of-command, and measure success.

**Fig. 1:**
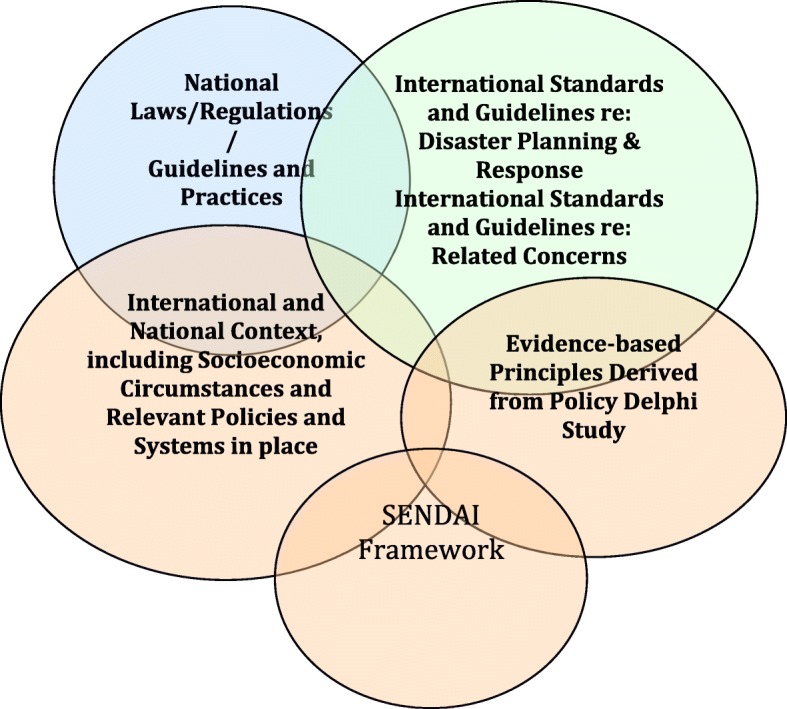
Graphic depiction of findings

Figure 1 presents a graphic depiction of the findings of this study, on which the following conclusions are based. It depicts the intersection among five domains:

1. International Standards and guidelines regarding disaster planning and response and related concerns (e.g., human rights, refugees, and climate change)
2. National Laws or Regulations, Guidelines and Practices relevant to disaster planning and response and related concerns (e.g., human rights and refugees)
3. National context, including socioeconomic circumstances and relevant policies and systems (e.g., governance, economic indicators, social support norms, health system, emergency response system, telecommunications, environmental/climate change circumstances and policies, water and sanitation, telecommunications, transportation, and public safety)
4. Evidence-based principles derived from this policy Delphi study: On-going and disaster-specific community engagement; proactive planning; addressing local and international complexities; considering and adhering to ethical and human rights accords, including balancing individual and societal rights; ensuring evidence-based policies and practices; information dissemination
5. SENDAI Framework: understanding disaster risk, strengthening disaster risk governance to manage disaster risk, investing in disaster risk reduction for resilience, enhancing disaster preparedness for effective response and to “build back better” in recovery, rehabilitation and reconstruction

The most effective means of facilitating the development and implementation of consistent, coordinated policies and practices within, between and among nation states to take into consideration the overlapping domains might be for the United Nations Office for Disaster Risk Reduction to take the lead in engaging key organizations in the necessary dialogue and collaboration. Relevant organizations would include, for example, related UN agencies, health professional associations, non-governmental and bilateral organizations that provide disaster responses; and national organizations with responsibility for disaster planning and response.

The United Nations Office for Disaster Risk Reduction could serve as the Secretariat for compiling and presenting evidence-based practices, as well as for the consideration of policies and practices based on this evidence. The focus should be on managerial, political and social feasibility of implementing the policies and practices, resource requirements (e.g., capacity-strengthening of response personnel, cost of equipment and supplies, development and implementation of information systems) and how to address them, and ethical and human rights implications. The United Nations Office for Disaster Risk Reduction could also provide a focal point for meetings to address evidence-based disaster planning and response.

## Conclusions

This study yielded three main goals as a means of improving the quality of disaster planning and response: 1) increased accessibility to scientific evidence, 2) improved communication among stakeholders, and 3) engagement of multiple beneficiaries to facilitate the development of evidence-based ‘best practices’ for disaster planning and response. Each of these goals can be implemented in ways that respect local culture and tradition while holding fast to the best tenets of evidence-based scientific practice. The consensus of respondents in this Delphi iterative study was optimistic for better future disaster plans and responses.

To improve evaluation of the effectiveness of pre-disaster and post-disaster planning and response, steps must be taken to plan the project with the desired outcome in mind and establish uniform methods of evaluation. Planning the project with the desired outcome in mind allows for a more structured approach with easily recognizable objectives, making the measuring of the success of these objectives much easier. This allows the evaluation to become a part of the planning process itself, ensuring that a proper evaluation is carried out and not merely an afterthought. A uniform method of evaluation will create a standard for the evaluation of disaster planning and response practices and activities, which will improve the ability to gather actual evidence on these practices.

Recent humanitarian disasters – due to natural and man-made hazards or a combination of the two – reinforce the need for more effective, efficient, humane responses at the local, national and international levels. This study has yielded findings that can be used to strengthen planning and response – taking into account evidence based on research that has been carried with the engagement of community members and with support by key stakeholders.

## Additional files

Additional file 1: Round 1. Questionnaire. (PDF 1403 kb)

Additional file 2: Round 2. Questionnaire. (PDF 119 kb)

## Abbreviations

DALYS: Disability Adjusted Life Years
GU: Georgetown University
IRB: Institutional Review Board
NGO: Non-Governmental Organization
QALYS: Quality adjusted Life Years
UN: United Nations
UNISDR: United Nations International Strategy for Disaster Reduction
WHO: World Health Organization
