# Supplementary material for: Improving the science and evidence base of disaster response: a policy research study
Source: BMC Health Serv Res. 2019 May 2;19:274. doi: 10.1186/s12913-019-4102-5 (PMC6498534; doi:10.1186/s12913-019-4102-5)
Supplement: Supplementary file 1 — Round 1. Questionnaire. (PDF 1403 kb) [file 12913_2019_4102_MOESM1_ESM.pdf]

EVIDENCE AID: Improving the Science and Evidence Base of Disaster Response: A POLICY DELPHI ENGAGEMENT

Conducted by a consortia of organizations comprising: Evidence Aid, Georgetown University, and the Uniformed Services University of the Health Sciences.

Participation in this study is entirely voluntary at all times. You can choose not to participate at all, decline to answer any of the questions, or discontinue participation and not submit the online survey. Regardless of your decision, there will be no effect on your relationship with the researchers or any other negative consequences. There are no risks associated with participation in this study. While you will not experience any direct benefits from participation, the information we collect may benefit others in the future by expanding knowledge regarding responses to disasters.

Your responses are anonymous; no unique identifying information is collected. If you provide your name and give us permission to do so, you may be listed in a final report as one of the respondents, but there will be no links with any of your responses. Once you submit your completed survey, there will be no way to withdraw from the study because the survey contains no identifying information. Your completion of the survey and submission through SurveyMonkey implies your consent to participate in the study. A brief summary of the findings of the first questionnaire will be sent to all those invited to participate at the same time that the second questionnaire is disseminated. They will also receive the study's final report. We will send this to everyone who is invited to participate because we will not be able to identify those who have completed the survey and those who have not done so.

Permission to conduct the study has been obtained from the Institutional Review Board of Georgetown University prior to any data collection; the IRB number is 2015-1357.

If you have any questions regarding Evidence Aid, please contact Professor Mike Clarke (m.clarke@qub.ac.uk) or Claire Allen (callen@evidenceaid.org). If you have any questions regarding this first questionnaire or this research study in general, please contact Professor Irene Jillson (iaj@georgetown.edu or by phone at 001-202-687-1312).

Round 1: Exploring the Issues

Demographics

1. Please indicate the type of organization in which you currently work *primarily* (that is, 50% or more of your time).

- [ ] Academic Institution/University
- [ ] National Government (if yes, select one below)
- [ ] - National Government: Health Agency
- [ ] - National Government: Military
- [ ] - National Government: Aid/International Development
- [ ] - National Government: Other (Please Specify Below)
- [ ] International Development
- [ ] Private Sector (if yes, select one below)
- [ ] - Private Sector: Non Profit Development Assistance
- [ ] - Private Sector: Non Profit Other (Please Specify Below)
- [ ] - Private Sector: Private (Please Specify Below)
- [ ] Other (Please Specify)

Demographics

2. Please specify your role; that is, the one in which you currently work*primarily* (that is, 50% or more of your
time).

- [ ] Administration/Management
- [ ] Consultant/Advisor
- [ ] Clinician (if yes, select one below)
- [ ] - Clinician: Physician
- [ ] - Clinician: Nurse
- [ ] - Clinician: Community Health Worker
- [ ] - Clinician: Laboratory Technician
- [ ] - Clinician: Other (Please Specify Below)
- [ ] Professor/Teacher
- [ ] Researcher
- [ ] Other (Please Specify)

3. Indicate the country in which your work is based; that is, where your office is located, not the headquarters of the organization for which you work, if it is different. (Drop-down menu)

Evidence For Best Practices in Disaster Response

4. On a scale of 1-6, with 6 being that you completely agree and 1 being that you completely disagree, indicate your view of the following statements and provide an explanation for your viewpoint. For each question item, if you believe that you do not have sufficient information to respond, select x (no answer) as your response.

The nature of the ‘evidence’ for disaster response is primarily ‘best practice’ information rather than research-based evidence. For purposes of this study, ‘research-based evidence’ is defined as that which is predicated on published research that has been independently reviewed and considered to be relevant, reliable and valid.

1 (Completely Disagree)	2	3	4	5	6 (Completely Agree)	N/A
◯	◯	◯	◯	◯	◯	◯

Explanation/Comment

5. Describe up to three ways in which research-based evidence regarding disaster response can be improved.

a.

b.

c.

Evidence For Best Practices in Disaster Response

6. The research-based evidence for disaster response is sufficiently sound to warrant its use as the basis for strategic planning by national governments.

1 (Completely Disagree)	2	3	4	5	6 (Completely Agree)	N/A
○	○	○	○	○	○	○

Explanation/Comment

Evidence For Best Practices in Disaster Response

7. The research-based evidence for disaster response is sufficiently sound to warrant its use as the basis for strategic planning by international development agencies.

1 (Completely Disagree)	2	3	4	5	6 (Completely Agree)	N/A
○	○	○	○	○	○	○

Explanation/Comment

Practical Use Of Evidence For Best Practices in Disaster Response

8. On a scale of 1-6, with 6 = completely agree and 1 = completely disagree, indicate your view of the following statements and provide an explanation for your viewpoint.

Cochrane-style systematic reviews (for examples, see www.cochranelibrary.com) are used in a strandardised way to synthesize evidence to inform contextually specific evidence of the effects of interventions, actions and strategies in disaster response.

1 (Completely Disagree)	2	3	4	5	6 (Completely Agree)	N/A
○	○	○	○	○	○	○

Explanation/Comment

Practical Use Of Evidence For Best Practices in Disaster Response

9. Cochrane-style systematic reviews (for examples, see www.cochranelibrary.com) **should be** used in a standardized way to synthesize evidence to inform contextually specific evidence of effectiveness in disaster response.

1 (Completely Disagree)	2	3	4	5	6 (Completely Agree)	N/A
○	○	○	○	○	○	○

Explanation/Comment

Practical Use Of Evidence For Best Practices in Disaster Response

10. It is possible for national governments to achieve better social return on investment in disaster response by implementing actions or interventions that are based on research evidence? For purposes of this study, ‘social return’ includes social, economic and health benefits to individuals, communities and the population of the affected country(ies).

1 (Completely Disagree)	2	3	4	5	6 (Completely Agree)	N/A
○	○	○	○	○	○	○

Explanation/Comment

11. If you believe it is possible, provide up to three examples of how this can be accomplished.

a.

b.

c.

12. If you do not believe this is possible, please explain why:

Practical Use Of Evidence For Best Practices in Disaster Response

13. It is possible for international multinational organizations to achieve better social return on investment in disaster response.

1 (Completely Disagree)	2	3	4	5	6 (Completely Agree)	N/A
○	○	○	○	○	○	○

Explanation/Comment

14. If you believe the above statement is possible, provide up to three examples of how this can be accomplished.

a.

b.

c.

15. If you do not believe this is possible, please explain why:

Practical Use Of Evidence For Best Practices in Disaster Response

16. It is possible for international non-profit organizations to achieve better social return on investment in disaster response.

1 (Completely Disagree)	2	3	4	5	6 (Completely Agree)	N/A
○	○	○	○	○	○	○

Explanation/Comment

17. If you believe the above statement is possible, provide up to three examples of how this can be accomplished.

a.

b.

c.

18. If you do not believe this is possible, please explain why:

Practical Use Of Evidence For Best Practices in Disaster Response

19. Describe up to three ways that knowledge and evidence derived from research could be used to inform decision-making in disaster response.

a.

b.

c.

Factors that Impact on Effective Disaster Response Decision-Making

Various frameworks for consideration of healthcare decision-making and responsible science – including application of science and technology to addressing health problems such as disaster relief exist, each of which includes multiple factors. This section addresses these factors with respect to responses to disasters.

20. Rank the top five factors in the order that you believe they impact on the effectiveness of disaster response, including responses by the affected country, donors and international NGOs that provide or channel resources. Using the following list, assign the number that corresponds to your ranking of the item: 1 = the most important factor, 2 = the next most important factor, and so on, until you have selected your top five. You may also add up to two factors for inclusion in your top five if you believe they are important but are not included in this list.

☐	a. Political influences of the government in which the disaster occurs
☐	b. Political influence of governments from outside the country in which the disaster occurs
☐	c. Political influences of international multinational organizations in the disaster response
☐	d. Political influences of bilateral donor agencies in the disaster response
☐	e. Political influences of non-profit organizations of the country in which the disaster occurs
☐	f. Political influences of non-profit organizations from outside the country in which the disaster occurs, which are engaged in disaster response
☐	g. Influence of for-profit organisations of the country in which the disaster occurs
☐	h. Influence of for-profit organisations from outside the country in which the disaster occurs, which are engaged in disaster response
☐	i. Economic influences of the country in which the disaster occurs
☐	j. Global economic influences in the country in which the disaster occurs
☐	k. Sociological trends in the country in which the disaster occurs
☐	l. International legal factors, including international agreements and regulations in the country in which the disaster occurs
☐	m. National legal factors, including for example laws and regulations regarding local NGOs; reconstruction; engagement of foreign clinicians in medical services in the country in which the disaster occurs

- [ ] n. Ethical factors, including international guidelines regarding health research in the context of disaster relief; allocation of resources; end-of-life decisions in the country in which the disaster occurs
- [ ] o. Post-colonial linkages between donor countries and countries affected by disasters
- [ ] p. Other Factor (please specify below in Q21)
- [ ] q. Other Factor (please specify below in Q21)

21. If you selected 'Other Factor', please specify below:

p. Other Factor: ____________________

q. Other Factor: ____________________

Factors that Impact on Effective Disaster Response Decision-Making

22. Indicate whether you believe each of your selected five factors has primarily positive or negative impact on the effectiveness of disaster response. If you believe it has both, indicate that.

	Primarily positive impact	Primarily negative impact	Both	N/A
a: Political influences of the government of the country in which the disaster occurs	○	○	○	○
b: Political influence of governments from outside the country in which the disaster occurs	○	○	○	○
c: Political influences of multinational organizations in the disaster response	○	○	○	○
d: Political influences of bilateral donor agencies in the disaster response	○	○	○	○
e: Political influences of non-profit organizations of the country in which the disaster occurs	○	○	○	○
f: Political influences of non-profit organizations from outside the country in which the disaster occurs, which are engaged in disaster response	○	○	○	○
g: Influence of for-profit organisations of the country in which the disaster occurs	○	○	○	○
h: Influence of for-profit organisations from outside the country in which the disaster occurs, which are engaged in disaster response	○	○	○	○
i: Economic influences of the country in which the disaster occurs	○	○	○	○

	Primarily positive impact	Primarily negative impact	Both	N/A
j: Global economic influences in the country in which the disaster occurs	◯	◯	◯	◯
k: Sociological trends in the country in which the disaster occurs	◯	◯	◯	◯
l: International legal factors, including international agreements and regulations that apply in the country in which the disaster occurs	◯	◯	◯	◯
m: National legal factors, including for example laws and regulations regarding local NGOs; reconstruction; engagement of foreign clinicians in medical services in the country in which the disaster occurs	◯	◯	◯	◯
n: Ethical factors, including international guidelines regarding allocation of resources related to disaster relief and the conduct of health research in the context of disaster relief	◯	◯	◯	◯
o: Post-colonial linkages between donor countries and countries affected by disasters	◯	◯	◯	◯
p: Other	◯	◯	◯	◯
q: Other	◯	◯	◯	◯

Factors that Impact on Effective Disaster Response Decision-Making

23. We are interested in your ideas on how the top two factors you identified above might be most effectively addressed in the context of disaster relief. What can governments and disaster-relief agencies do to make the best use of the positive aspects of the factor and to ameliorate the negative aspects of the factor? For example, how can international regulations regarding research ethics in the context of disaster relief be most effectively utilized? How can the potential for competition for funding among international relief organizations be addressed? If you have evidence for how these approaches have worked, provide at least one citation or link.

Factor 1: (Write in the letter to identify the top factor you have chosen):

Making effective use of positive aspects, if any:

Addressing negative aspects, if any:

Factor 2 : (Write in the letter to identify the second top factor you have chosen):

Making effective use of positive aspects, if any:

Addressing negative aspects, if any:

Other comments:

Other

Thank you for taking part in this study. If you would like more information about Evidence Aid, or would like to sign up for the Evidence Aid newsletter, go to http://www.evidenceaid.org, follow us on Twitter using @EvidenceAid, or join our Facebook Group or 'like' our Facebook page.

24. If you would like to suggest questions for inclusion in the second round of this policy Delphi study, add up to three here.

a.

b.

c.
