# Supplementary material for: Improving the science and evidence base of disaster response: a policy research study
Source: BMC Health Serv Res. 2019 May 2;19:274. doi: 10.1186/s12913-019-4102-5 (PMC6498534; doi:10.1186/s12913-019-4102-5)
Supplement: Supplementary file 2 — Round 2. Questionnaire. (PDF 119 kb) [file 12913_2019_4102_MOESM2_ESM.pdf]

EVIDENCE AID: Improving the Science and Evidence Base of Disaster Response
A POLICY DELPHI ENGAGEMENT

Conducted by a consortia of organizations comprising:
Evidence Aid
Georgetown University
Uniformed Services University of the Health Sciences
Pan American Health Organization

Round 2: Research, Evidence, Policy and Practice: Exploring the Links

Informed Consent:

Participation in this study is entirely voluntary at all times. You can choose not to participate at all, decline to answer any of the questions, or discontinue participation and not submit the online survey. Regardless of your decision, there will be no effect on your relationship with the researchers or any other negative consequences. There are no risks associated with participation in this study. While you will not experience any direct benefits from participation, the information we collect may benefit others by expanding knowledge regarding responses to disasters. Your responses are anonymous; no unique identifying information is collected. If you provide your name and give us permission, you may be listed in a final report as one of the respondents, but there will be no links with any of your responses. When you have submitted your completed survey, there will be no way to withdraw your responses from the study because the survey contains no identifying information. Your completion of the survey and submission through SurveyMonkey implies your consent to participate in the study. A brief summary of the findings of this questionnaire (the second “round”) will be sent to all those invited to participate. They will also receive the study’s final report. We will send this to everyone who is invited to participate because we will not be able to identify those who complete the survey and those who do not do so. Permission to conduct the study has been obtained from the Institutional Review Board of Georgetown University prior to any data collection; the IRB number is 2015-1357.

A. Demographics

1. Please indicate the type of organization in which you currently work *primarily* (that is, 50% or more of your time).

- ☐ Academic Institution/University
- ☐ National Government (if yes, select one below)
- ☐ - National Government: Health Agency
- ☐ - National Government: Military
- ☐ - National Government: Aid/International Development
- ☐ - National Government: Other (Please Specify Below)
- ☐ International Development (non-government)
- ☐ Private Sector (if yes, select one below)
- ☐ - Private Sector: Non Profit, Development Assistance
- ☐ - Private Sector: Non Profit, Humanitarian Aid
- ☐ - Private Sector: Non Profit, Other (Please Specify Below)
- ☐ - Private Sector: Private (Please Specify Below)
- ☐ Other (Please Specify)

A. Demographics

2. Please specify your role; that is, the one in which you currently work *primarily* (that is, 50% or more of your time).

- ☐ Administration/Management
- ☐ Consultant/Advisor
- ☐ Clinician (if yes, select one below)
- ☐ - Clinician: Physician
- ☐ - Clinician: Nurse
- ☐ - Clinician: Community Health Worker
- ☐ - Clinician: Laboratory Technician
- ☐ - Clinician: Other (Please Specify Below)
- ☐ Emergency Responder (e.g., Firefighter, Emergency Medical Technician)
- ☐ Professor/Teacher
- ☐ Researcher
- ☐ Other (Please Specify)

3. Indicate the country in which your work is based; that is, where your office is located, not the headquarters of the organization for which you work, if it is different. (Drop-down menu)

B. Evidence for Best Practices in Disaster Planning and Response

4. What do you believe are the three most effective ways to improve the applicability of research-based evidence for *pre-disaster planning*? (Select the top three and comment)

- [ ] a. Ensure that research related to pre-disaster planning is decision-linked (i.e., fit for purpose).
- [ ] b. Ensure that potential users of research related to pre-disaster planning (policy-makers, planners, emergency response personnel) are involved in the design and/or implementation of the research.
- [ ] c. Ensure that multiple research methods are used (e.g., quantitative, qualitative, mixed method, economic analysis).
- [ ] d. Ensure that comprehensive research is carried out (e.g., addressing socioeconomic, life sciences, One Health, governance and other aspects of pre-disaster planning).
- [ ] e. Conduct research primarily that is narrowly focused, for example, with respect to certain medical-related trauma (e.g., amputations, infectious diseases following disasters, mental health consequences).
- [ ] f. Ensure the research is guided by priority setting or gap map exercises conducted with a robust methodology.
- [ ] g. Other (please specify below)
- [ ] h. Other (please specify below)

Other Suggestions/Comments:

5. What do you believe are the three most effective ways to improve the applicability of research-based evidence for *post-disaster response*? (Select the top three and comment)

- [ ] a. Ensure that research related to post-disaster planning is decision-linked (i.e., fit for purpose).
- [ ] b. Ensure that potential users of research related to post-disaster planning (policy-makers, planners, emergency response personnel) are involved in the design and/or implementation of the research.
- [ ] c. Ensure that multiple research methods are used (e.g., quantitative, qualitative, mixed method, economic analysis).
- [ ] d. Ensure that comprehensive research is carried out (e.g., addressing socioeconomic, life sciences, One Health, governance and other aspects of pre-disaster planning).
- [ ] e. Conduct research primarily that is narrowly focused, for example, with respect to certain medical-related trauma (e.g., amputations, infectious diseases following disasters, mental health consequences).
- [ ] f. Ensure the research is guided by priority setting or gap map exercises conducted with a robust methodology.
- [ ] g. Other (please specify below)
- [ ] h. Other (please specify below)

Other Suggestions/Comments:

B. Evidence for Best Practices in Disaster Planning and Response

6. Describe how you believe the following potential beneficiaries of disaster-related research should be engaged in research related to disaster planning and response, considering the lifespan of research from selecting priorities through designing and implementing the research to disseminating its findings. Describe briefly for each category of consumer and stakeholder.

a. Policy Makers:

b. Planners:

c. Responders (e.g., emergency medical personnel, other emergency responders (e.g., fire fighters, police), food and supply distribution personnel/volunteers, emergency utility and water and sanitation personnel, physical infrastructure):

d. Government officials from relevant ministries:

e. Other public employees (e.g., school teachers):

f. Local non-governmental organizations (e.g., those involved in houses of worship, community organizations):

g. Those affected:

h. Members of the public generally:

i. Other category (please specify):

7. Describe how you believe the following potential beneficiaries of disaster-related research should be engaged in applying research findings related to disaster planning and response to strengthening their capacity to plan for and respond to disasters. Describe briefly for each category of consumer and stakeholder.

a. Policy Makers:

b. Planners:

c. Responders (e.g., emergency medical personnel, other emergency responders (e.g., fire fighters, police), food and supply distribution personnel/volunteers, emergency utility and water and sanitation personnel, physical infrastructure):

d. Government officials from relevant ministries:

e. Other public employees (e.g., school teachers):

f. Local non-governmental organizations (e.g., those involved in houses of worship, community organizations):

g. Those affected:

h. Members of the public generally:

i. Other category (please specify):

B. Evidence For Best Practices in Disaster Planning and Response

8. Poor availability of research-based evidence related to disaster planning and response has been identified as a problem. What are the three most effective ways to improve the availability of research-based evidence? (Select the top three and comment)

- ☐ a. Dissemination of all research-based findings related to disaster planning and response through a central, global network.
- ☐ b. Dissemination of research-based findings related to disaster planning and response through a central, global network **only if they have been critically reviewed by an approved body.**
- ☐ c. Dissemination of all research-based findings related to disaster planning and response through international and national associations of related professionals (e.g., World Medical Association, International Nurses' Association, International Association of Emergency Managers).
- ☐ d. Dissemination of all research-based findings related to disaster planning and response through international and national associations of related professionals (e.g., World Medical Association, International Nurses' Association, International Association of Emergency Managers) **only if they have been critically reviewed by an approved body.**
- ☐ e. Dissemination of all research-based findings related to disaster planning and response through the relevant UN institutes.
- ☐ f. Dissemination of all research-based findings related to disaster planning and response through one UN institute and critically reviewed by the same institute.
- ☐ g. Ensuring that research-based evidence is available at no or minimal cost.
- ☐ h. Other (please specify below)
- ☐ i. Other (please specify below)

Other Suggestions/Comments:

9. Given that the use of ‘best practice information’ (that is, information that is more experiential than research-based) may be more commonly used than ‘research-based’ evidence, what are the three most effective ways to ensure that these best practices inform the design of disaster-related research to prove evidence of effectiveness? (Select the top three and comment)

- ☐ a. Dissemination of ‘best practice information’ related to disaster planning and response through a central, global network.
- ☐ b. Dissemination of ‘best practice information’ related to disaster planning and response through a central, global network **only if they have been critically reviewed by an approved body.**
- ☐ c. Dissemination of ‘best practice information’ related to disaster planning and response through international and national associations of related professionals (e.g., World Medical Association, International Nurses’ Association, International Association of Emergency Managers).
- ☐ d. Dissemination of ‘best practice information’ related to disaster planning and response through international and national associations of related professionals (e.g., World Medical Association, International Nurses’ Association, International Association of Emergency Managers) **only if they have been critically reviewed by an approved body.**
- ☐ e. Dissemination of all research-based findings related to disaster planning and response through the relevant UN institutes.
- ☐ f. Dissemination of all research-based findings related to disaster planning and response through one UN institute and critically reviewed by the same institute
- ☐ g. Ensuring that research-based evidence is available at no or minimal cost
- ☐ h. Other (please specify below)
- ☐ i. Other (please specify below)

Other Suggestions/Comments:

B. Evidence for Best Practices in Disaster Planning and Response

10. Should evidence regarding disaster planning and response be used only if it is peer reviewed, published and critiqued?

- [ ] a. Peer Reviewed
- [ ] b. Published
- [ ] c. Critiqued
- [ ] d. All of the above
- [ ] e. No, it should always be published

Comment:

B. Evidence for Best Practices in Disaster Planning and Response

For each of the expected outcomes of the UNISDR Road Map for Implementation of Sendai, please describe one challenge to achieving the outcome and one approach to addressing and overcoming that challenge.

11. Assess and update the current state of data, scientific and local and indigenous knowledge and technical expertise availability on disaster risks reduction and fill the gaps with new knowledge.

Challenge:

Approach to Overcoming the Challenge:

12. Synthesize, produce and disseminate scientific evidence in a timely and accessible manner that responds to the knowledge needs of policy-makers and practitioners.

Challenge:

Approach to Overcoming the Challenge:

13. Ensure that scientific data and information support are used in monitoring and reviewing progress towards disaster risk reduction and resilience building.

Challenge:

Approach to Overcoming the Challenge:

14. Build capacity to ensure that all sectors and countries have access to, understand and can use scientific information for better informed decision-making.

Challenge:

Approach to Overcoming the Challenge:

Evidence for Best Practices in Disaster Planning and Response

For each of the expected outcomes of the UNISDR Road Map for Implementation of Sendai, please describe <u>one challenge</u> to achieving the outcome and <u>one approach</u> to addressing and overcoming that challenge.

15. Support a stronger involvement and use of science to inform policy- and decision-making within and across all sectors at all levels.

Challenge:

Approach to Overcoming the Challenge:

16. Provide scientific evidence to enable decision-making of policy options for investment and development planning vulnerable communities and locations.

Challenge:

Approach to Overcome the Challenge:

17. Identify and respond to the needs of policy- and decision-makers at all levels for scientific data and information to strengthen preparedness, response and to "Build Back Better" in Recovery, Rehabilitation and Reconstruction to reduce losses and impact on the most vulnerable communities and locations.

Challenge:

Approach to Overcome the Challenge:

B. Evidence for Best Practices in Disaster Planning and Response

18. Provide a specific example each for ethical, legal and social issues in disaster planning and response, and provide an example of:
a) ways that the issue has been adequately addressed
b) ways that the issue could be addressed more effectively through improved evidence.

Ethical example

a)

b)

Legal example

a)

b)

Social example

a)

b)

19. What is the most effective way for best practices and improved evidence to be used to help ensure co-ordination and co-operation (and to reduce competition) among stakeholders in the following categories:

a. Between public and private sectors in the country in which the disaster occurs:

b. Within the public sector in the country in which the disaster occurs:

c. Within the private sector (including non-profit NGOs) in the country in which the disaster occurs, between and among international and regional multilateral agencies:

d. Among Donors:

20. What is the most effective way that best practices and improved evidence can be used to help ensure accountability for disaster planning and response?

Thank you for taking part in this study. If you would like more information about Evidence Aid, or would like to receive the Evidence Aid newsletter, go to www.evidenceaid.org. You can also follow us on Twitter (@EvidenceAid,) join our Facebook Group or 'like' our Facebook page. If you have any questions related to this policy Delphi, please contact Professor Irene Jillson at iaj@georgetown.edu
